# Utilization of mental health services in relation to the intention to reduce chemsex behavior among clients from an integrated sexual health services center in Taiwan

**DOI:** 10.1186/s12954-023-00777-y

**Published:** 2023-04-16

**Authors:** Yu-Ru Hung, Tsan-Tse Chuang, Tsai-Wei Chen, An-Chun Chung, Meng-Tzu Wu, Su-Ting Hsu, Nai-Ying Ko, Carol Strong

**Affiliations:** 1 Department of Public Health, College of Medicine, National Cheng Kung University, 8F-8068, No. 138, ShengLi Rd., North Dist., Tainan City, 704 Taiwan; 2 Taiwan Love and Hope Association, Kaohsiung, Taiwan; 3 Healing, Empowerment, Recovery of Chemsex (HERO) Health Center, HÉROS, Kaohsiung, Taiwan; 4 Department of Community Psychiatry, Kaohsiung Municipal Kai-Syuan Psychiatric Hospital, Kaohsiung, Taiwan; 5 Department of Nursing, College of Medicine, National Cheng Kung University, Tainan, Taiwan

**Keywords:** Chemsex, Sexualized drug use, Chemsex-care services, Harm reduction services, Service utilization, GBMSM

## Abstract

**Introduction:**

The intention of chemsex-practicing gay and bisexual men and other men who have sex with men (GBMSM) to reduce their drug use is an important factor for the utilization of harm reduction services. This study aimed to examine data from an integrated sexual health services center to understand the relationship between the intention to reduce chemsex behavior and chemsex-related utilization of mental health services among GBMSM who engage in chemsex.

**Method:**

We used data collected from Healing, Empowerment, Recovery of Chemsex (HERO), an integrated health center in Taiwan, between November 2017 and December 2021. As the baseline, clients were asked to rate the current and ideal proportions of their sexual activities that involved the use of MDMA, ketamine, methamphetamine, GHB/GBL, or mephedrone. Having the intention to reduce chemsex was defined as having a lower proportion of ideal engagement compared to actual engagement. The data on the use of the services provided at HERO were linked to the survey responses and compared to information gathered during regular follow-up visits. Univariable and multivariable logistic regression analyses and a Poisson regression analysis were performed on the data.

**Results:**

A total of 152 GBMSM reported engaging in chemsex, of whom 105 (69.1%) expressed the intention to reduce their chemsex behavior. Service utilization ranged from 23.0% for participating in meetings of a chemsex recovery group, 17.1% for visiting a mental health clinic, and 10.5% for using both of these services. The intention to reduce chemsex behavior significantly associated with visiting a mental health clinic (aOR = 4.68, *p* < 0.05), but its association with attending meetings of a chemsex recovery group was only marginally significant (aOR = 2.96, *p* < 0.1). Other factors that remained significantly associated with service use were a high frequency of substance use and living with HIV.

**Conclusion:**

Comprehensive harm reduction strategies, which touch on mental health, drug use management and recovery, are needed for those who want to reduce their chemsex behavior. Public health practitioners should endeavor to raise awareness of resources that are available for people who engage in chemsex and to minimize the barriers blocking their access to the appropriate services.

## Introduction

Chemsex refers to the use of drugs in a sexual context, typically by gay and bisexual men and other men who have sex with men (GBMSM). The term chemsex was first used in the UK and has gained attention on several continents in the past decade, including the USA, Australia, and parts of Europe [1–4]. The motivation for engaging in chemsex often includes the desire to enhance the sexual experience and to improve sexual performance [5]. Some of the most popular drugs used in chemsex include methamphetamine, gamma-hydroxybutyrate/gamma-butyrolactone (GHB/GBL), and mephedrone [6], but other substances such as cocaine [7], ketamine [8], and methylenedioxymethamphetamine (MDMA) [9] are also reported. The rise of this increasingly common practice has occurred alongside a boom in the popularity of dating applications on mobile phones, which facilitate meeting potential sex partners and accessing drugs [10]. The prevalence of chemsex in Asia is estimated at 11.1–11.8% among the general population of men who have sex with men (MSM) in Thailand, Hong Kong, and Malaysia [11–13], and it is estimated to be considerably higher among MSM living with HIV, at 38.9% in Taiwan [14] and 44.2% in Hong Kong [15]. It has been suggested that a large proportion of MSM who have engaged in chemsex in Taiwan have used methamphetamine at some point in their lives (27.1%), and among them 46% showed signs of methamphetamine dependency [5].

When the use of drugs becomes problematic, such that users lose control of aspects of their lives such as work, finances, social relationships, or the ability to adopt a healthier lifestyle [16], it is important to carry out interventions or harm reduction measures in order to help users quit or better control the harm caused by their use of drugs. It has been recommended that healthcare service providers and their clients, in their attempts to devise harm reduction measures to cope with the adverse effects of chemsex, consider three possible avenues: HIV-focused schemes, drug-focused schemes and sex-focused schemes, with some overlap among these schemes and the corresponding harm reduction options [17]. Some of the components of a comprehensive program aimed at drug-related harm reduction, which have been endorsed by the World Health Organization (WHO), include needle and syringe programs, opioid substitution therapy, HIV testing and counseling, antiretroviral therapy (ART), and targeted Information, Education and Community (IEC) efforts for intravenous drug users and sexual partners [18]. It should be noted that while these components are not specific to chemsex, they still form the basis of many common chemsex resources available for GBMSM, such as providing sterile needles and other injecting equipment [19], sexually transmitted infections (STI)/HIV care and screening [20], and referral to substance use services [19, 21].

The intention to achieve behavioral change or to get help in the context of drug use problems has been shown to be an important indicator of the use of health services by such populations as cocaine users, and the motivation to change seems to have a significant effect on one’s attendance at self-help group meetings [22]. To the best of our knowledge, however, the focus of the research on such issues has rarely been placed on the subject of chemsex. Therefore, there is a lack of research on how many GBMSM exhibit the intention to change their chemsex behavior and on how this is related to the actual service utilization, particularly mental health services.

Priority should be given to mental health considerations and drug use management in the provision of services for people who engage in chemsex. Mental health concerns such as depression, anxiety, and psychotic symptoms have been observed among those who practice chemsex [23–25]. For example, among MSM who engage in chemsex in Germany, the prevalence of depression has been found to be 11.9%, while for somatization it was 13.5%, and for generalized anxiety disorder it was 8.3%—all of which were higher than the rate for the German population in general [26]. It has also been found that chemsex-practicing MSM in Singapore are more likely than those who do not engage in chemsex to report high rates of depression and more instances of suicidal ideation [24]. Data on the situation in Taiwan show that methamphetamine dependency is associated with feelings of loneliness among GBMSM who engage in chemsex [5]. Finally, in Malaysia, suicidal ideation, depression, paranoia, and hallucinations are some of the health problems reported by GBMSM who engage in chemsex [27].

However, to the best of our knowledge, the mental healthcare needs of people engaging in chemsex are not being adequately met. A qualitative study of the service needs assessments provided to GBMSM who use methamphetamine in a sexual context found that these people came up against barriers and faced limited options when attempting to access mental health support services, unless they had reached a relatively severe state of mental illness [28]. In many countries, chemsex-care services are available, such as Lighthouse Social Enterprise in Vietnam [29], ACON and Thorne Harbour Health in Australia [3, 30], and Mainline in the Netherlands [31]. However, the utilization at which mental health services are used is less often mentioned compared to HIV-related services. For example, the rate of HIV screening performed with GBMSM who engage in chemsex has been found to range from 55% in Malaysia [32] to 80.5% in Hong Kong [33], whereas an online survey conducted in Germany reported that only 30% of the people who engage in chemsex had previously received psychotherapeutic treatment [34].

The present study aimed to elucidate the relationship between the intention to change chemsex behaviors and chemsex-care service utilization, specifically mental healthcare services. We used data from a sample of chemsex-practicing GBMSM who visited an integrated sexual health services center between 2017 and 2021. We hypothesized that the people who expressed the intention to reduce their chemsex behavior would have higher service utilization in chemsex-care services by the end of 2021.

## Program description

The Healing, Empowerment, Recovery of Chemsex (HERO) center is a one-stop integrated sexual health and substance use services center that applies harm reduction principles to its approach to treatment. It was established in Kaohsiung City, in southern Taiwan, in November 2017. HERO was founded by the Taiwan Love and Hope Association, a non-governmental organization and community health center that advocates HIV prevention and treatment and provides help for GBMSM. It has a decades-long relationship with the local LGBT community.

HERO aims to come to the aid of people who engage in chemsex by providing an empowering therapeutic environment, which activates their inner healing powers in order to help them to recover from drug use. The center provides prevention and treatment services for people at risk of HIV/STI, including testing, counseling, pre-exposure prophylaxis (PrEP) and post-exposure prophylaxis (PEP) for HIV prevention, and chemsex-care services. In 2022, the HÉROS health center evolved from the original HERO center. HÉROS combines a community drugstore, a clinic, and a community health center and aims to facilitate access to chemsex-care services for GBMSM who engage in chemsex.

**Fig. 1:**
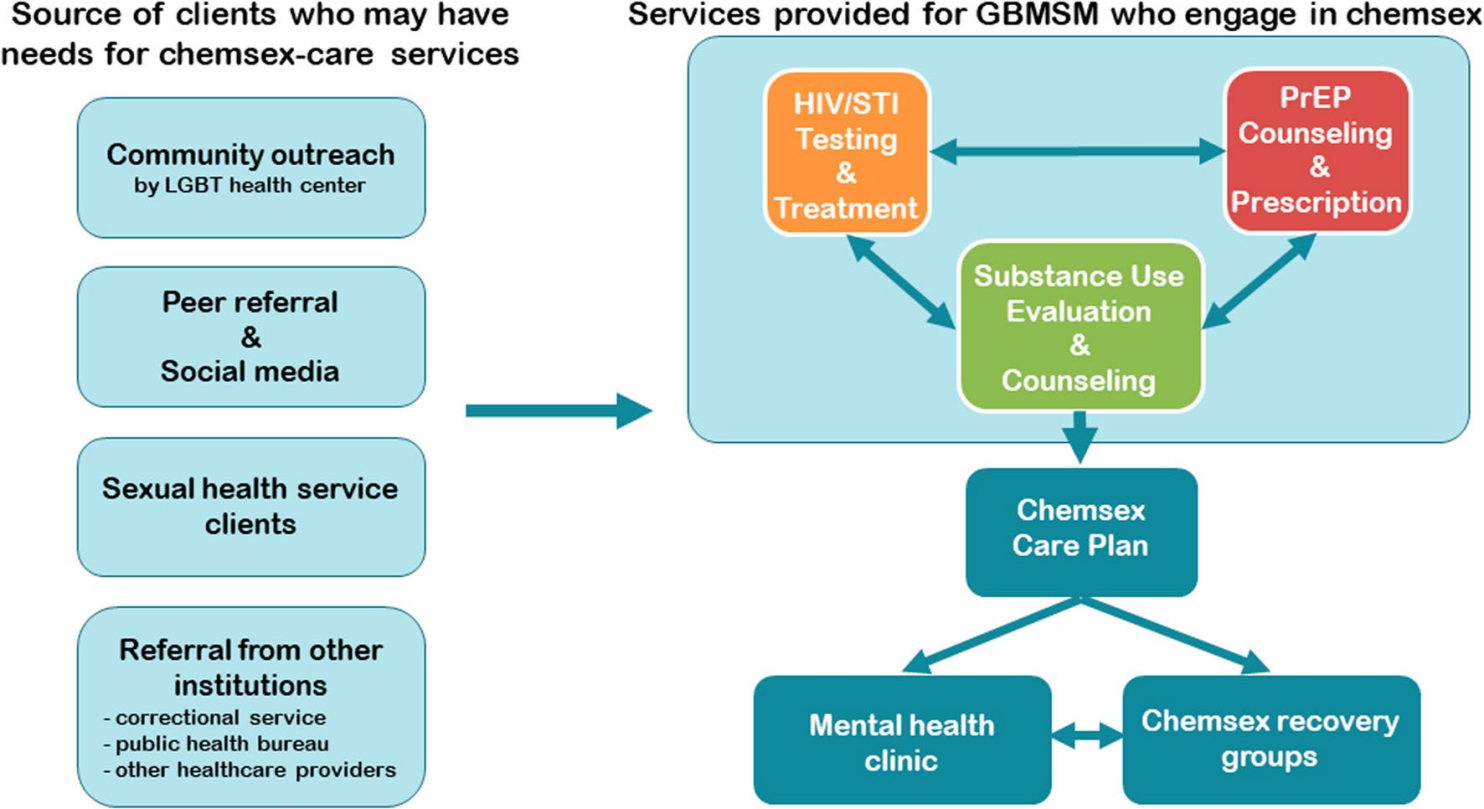
Flowchart of the services provided by HERO for GBMSM who engage in chemsex

Due to the wide array of sexual health services provided by HERO, their clientele includes people outside the GBMSM who engaged in chemsex. Figure 1 shows not only the chemsex-care services offered at HERO but also the variety of clients who have used these services or who have been deemed in need of such services after undergoing an evaluation. The clients of HERO arrive at the center in different ways. Some cases are the result of community outreach efforts: People who potentially need chemsex-care services are referred to HERO by NGOs and community health centers that cater for the local LGBT community. A second source of clients at HERO is peer referral: People find out about HERO from other people such as the partners and friends of those who have used HERO services, and from postings on social media about events and services offered at HERO. Thirdly, the need for chemsex-care services is sometimes discovered during their visit at HERO. For example, people who undergo HIV/STI testing and treatment may reveal chemsex behavior during their conversations with social workers, physicians, and other healthcare workers, eventually leading to a referral for chemsex-care services. The last major source is referral from correctional service, the public health bureau, or other healthcare providers.

### HERO provides three major chemsex-care services

As mentioned above, Fig. 1 shows a flowchart of the services provided by HERO for GBMSM who practice chemsex. As clients of the center, they have access to HIV/STI testing and treatment as well as PrEP counseling and prescriptions if needed. They are also evaluated and counseled for their substance use on the basis of the Chemsex Care Plan. Finally, when the need presents itself, they are advised to visit a mental health clinic or to join chemsex recovery groups.

#### The chemsex care plan

The Chemsex Care Plan was developed by David Stuart [35] based on motivational interviewing techniques in order to help GBMSM understand their goals and their motivation or lack of motivation with regard to changing their chemsex behavior. The impetus for this program was a desire to help healthcare workers to encourage their clients to make more honest disclosures while assessing their sexual history [36]. The Chemsex Care Plan provides a safe environment in which to raise the clients’ awareness both of their harmful behavior and of the options open to them in order to help them more clearly understand their current situation and thus decide whether they need the relevant services [36]. The fundamental technique employed in the context of this program, motivational interviewing, allows clients to feel reluctant or ambivalent about making behavioral changes until they are completely ready to do so. Consequently, a key feature of the program is the freedom it grants clients to express defensiveness and to remain in a state of denial while at the same time being encouraged to choose a goal to work toward, such as abstinence, caution, or taking a short break away from the harmful behavior [36]. The Chemsex Care Plan is typically used with new members in a recovery group, but not limited to meeting participants, and it can be repeated afterward as required. The one-on-one interview is carried out by a staff member in a consultation room. Agreement between the client and the staff member results in a care plan tailored to the client’s level of motivation and confidence with regard to changing their chemsex behavior, with possible outcomes ranging from complete abstinence to exercising caution to refusing to change.

#### Mental health services and treatment

The mental health clinic at HERO provides services once a week. All new members of chemsex recovery groups are encouraged to make an appointment with a psychiatrist and also to schedule follow-up visits every 3–6 months. If clients require access to legal resources and consultations, the social worker at HERO can refer them to government-funded projects that provide legal help. The main benefit of having a mental health clinic at HERO for clients who engage in chemsex is to provide an immediate link to HIV-related services such as treatment, testing, and PrEP.

#### Chemsex recovery group

Meetings of chemsex recovery groups have been led by psychologists, starting at once per month in 2017 and increasing to twice monthly since 2019. Techniques that are employed at the meetings include self-exploration, mindfulness-based relapse prevention, and self-regulated learning.

## Methods

### Study design and participants

For this cohort study, the participants were individuals who approached HERO for chemsex- or HIV/STI-related services between November 2017 and December 2021. All visitors to the center were invited to take part in the study, including individuals who were there for HIV and STI testing and treatment, PrEP counseling and prescription, substance use evaluation and counseling, and other chemsex-care services. Participation was voluntary and not compensated. After consenting to the conditions of the study, participants were asked to complete a baseline questionnaire during their initial visit in order to collect information on sociodemographic characteristics, substance use, diagnosis of HIV/STIs, PrEP use, and mental health. They were also given follow-up questionnaires during subsequent visits. However, the information gathered with follow-up questionnaires did not factor into the statistical analyses. The criteria for eligibility included being a member of the GBMSM community, being at least 20 years of age, and practicing chemsex, as determined based on responses to the baseline questionnaire. In Taiwan, research ethics require individuals to be at least 20 years of age to provide consent for participation in a study without parental approval. Questionnaire responses were linked to clinical data recorded by HERO staff regarding the participants use of the services available at HERO. The study protocol was reviewed and approved by the Institutional Review Board of the National Cheng Kung University Hospital [#B-BR-106-046].

### Measurement of outcomes and variables

The primary outcome of interest was chemsex-related mental health service utilization, including: (1) a history of having attended meetings of a chemsex recovery group, (2) a history of having visited a mental health clinic, and (3) the types of services that participants used which could include the recovery group meetings and the visits to a mental health clinic. The relevant data were retrieved from the programmatic records which are kept at HERO.

On a visual analogue scale ranging from 0 to 100% included in the baseline questionnaire, participants were asked to rate the proportion of their sexual activities that involved the use of MDMA, ketamine, methamphetamine, GHB/GBL, or mephedrone. The corresponding questionnaire items were: (1) “What percentage of your *current* sex life involves chemsex?” and (2) “*Ideally*, what percentage of your sex life do you wish included chemsex?” The *intention to reduce chemsex behavior* was defined as a participant wanting a lower proportion of chemsex in his current sex life.

The sociodemographic data were age, gender, monthly income, educational level, job status, and relationship status. We dichotomized the variables of age (< 35 vs. ≥ 35), monthly income (≤ 30,000 New Taiwan Dollar (NTD; 1 USD ~  = 29 NTD) vs. > 30,000 NTD), educational level (high school degree or below vs. bachelor's degree or above), and job status (unemployed vs. employed). The variable of relationship status was divided into single, regular partners, and casual partners.

Participants were asked to report whether they had used any substances in the previous year by selecting items from a list of 14 options: alcohol, methamphetamine, MDMA, GHB/GBL, ketamine, mephedrone, marijuana, sedative, alkyl nitrites (also known as ‘poppers’ and ‘Rush’), cocaine, Viagra, lysergic acid diethylamide (LSD), psilocybin mushrooms, and “coffee-pack” (a mixture of substances packaged as a coffee pack without listing the ingredients). Poly-drug use was defined as the use of more than two of the substances on this list in the previous year. Frequency of substance use in the previous year was reported on a scale with the following options: never, seldom, sometimes, usually, and always. The answers were dichotomized into low frequency (never, seldom, or sometimes) and high frequency (usually or always).

The questionnaire also addressed HIV status. Participants were asked about their current HIV status (i.e., living with HIV vs. not living with HIV) when they first joined this study.

To evaluate the participants’ degree of anxiety, seven questions were adopted from the Generalized Anxiety Disorder-7 (GAD-7) assessment scale [35]. Participants were asked to indicate how often they had been bothered by the following problems in the previous two weeks: feeling nervous, anxious or on edge; and not being able to stop or control worrying. They responded on a scale of 0 (not at all) to 3 (nearly every day). As a measure of depression, the Patient Health Questionnaire-9 (PHQ-9) assessment scale was used [37]. Participants were asked to indicate how often they had been bothered by the following problems in the previous two weeks: little interest or pleasure in doing things; poor appetite; and overeating. They again responded on a scale of 0 (not at all) to 3 (nearly every day). The total scores on both the GAD-7 and the PHQ-9 were dichotomized on the basis of the cutoff value of 10 [38]. However, due to highly overlapping participants lacking data on the GAD-7 and PHQ-9 and for the sake of brevity, a combined variable was created. Thus, the participants were divided into two groups: those facing a slight risk of depression and anxiety (i.e., the total score on both scales ≤ 10) and those at moderate-to-high risk of either depression or anxiety (i.e., the total score on at least one scale > 10).

### Statistical analyses

All statistical analyses were performed using R, Version 4.1.3 [39]. Descriptive statistics were computed to demonstrate the frequency and distribution of the occurrence of the various participant characteristics. Chi-squared tests were used to compare participants in terms of the intention to reduce chemsex behavior. Bar graphs were used to visualize the relationship between substance use and the intention to reduce chemsex behavior, as well as that between substance use and the use of chemsex-care services. We also used the Chi-squared test to examine whether participants took part in chemsex recovery group meetings and whether they used the services of a mental health clinic. In univariable and multivariable analyses, logistic and Poisson regressions were used to test for relationships between the use of these healthcare services and all other variables. The variables for which we obtained a *p*-value of less than 0.05 in the univariable models were then entered into the multivariable regression models, along with age and monthly income.

## Results

### Study participants

Of the 1215 participants who visited HERO for various services, including but not limited to HIV testing and treatment as well as PrEP counseling and prescriptions, and who completed the baseline questionnaire between November 2017 and December 2021, 152 (12.5%) were chemsex-practicing GBMSM who made up the sample on which the analyses were performed. Table 1 presents the baseline characteristics of the participants, which include: a median age of 28 years (IQR: 25–34.25); more than half (53.3%) with a monthly income of less than 30,000 NTD; 80.9% with a bachelor’s degree or higher; and 71.7% with jobs. More than half (52.0%) of the participants reported being single, whereas 32.2% reported having regular partners. A total of 36.2% of the participants reported poly-drug use in the previous 12 months, and 16.4% claimed a high frequency of substance use. More than a quarter (25.7%) of the participants reported living with HIV. Exactly one quarter (25.0%) of the participants showed symptoms of anxiety, 29.3% had symptoms of depression, and 32.1% had experienced episodes of either anxiety or depression in the previous two weeks.

**Table 1:** Baseline characteristics comparing participants with or without intention to reduce chemsex behavior (*N* = 152)

	Total *N* = 152	With intention to reduce *n* = 105 (69.1%)	Without intention to reduce *n* = 47 (30.9%)	*p*-value
	*N* (%)	*n* (%)	*n* (%)	
Age < 35	114 (75.0)	81 (77.1)	33 (70.2)	0.362
Monthly income ≤ NTD$30,000	81 (53.3)	63 (60.0)	18 (38.3)	0.013
Education ≤ bachelor’s degree	123 (80.9)	91 (86.7)	32 (68.1)	0.007
Unemployed	43 (28.3)	35 (33.3)	8 (17.0)	0.039
Relationship status				0.013
Single	79 (52.0)	57 (54.3)	22 (46.8)	
Regular partners	49 (32.2)	27 (25.7)	22 (46.8)	
Casual partners	24 (15.8)	21 (20.0)	3 (6.4)	
Poly-drug use	55 (36.2)	44 (41.9)	11 (23.4)	0.028
High frequency of substance use	25 (16.4)	17 (16.2)	8 (17.0)	0.898
Living with HIV	39 (25.7)	31 (29.5)	8 (17.0)	0.103
GAD-7 score > 10^a^	35 (25.0)	26 (26.8)	9 (20.9)	0.459
PHQ-9 score > 10^a^	41 (29.3)	31 (32.0)	10 (23.3)	0.297
GAD-7 and PHQ-9 scores at least one > 10^a^	45 (32.1)	34 (35.1)	11 (25.6)	0.268

NTD = New Taiwan Dollar; HIV = human immunodeficiency virus; GAD-7 = Generalized Anxiety Disorder-7; PHQ-9 = Patient Health Questionnaire-9

^a^12 Missing

Of the 152 participants, 105 (69.1%) expressed the intention to reduce their chemsex behavior on the baseline questionnaire. This tendency significantly correlated with a monthly income of less than 30,000 NTD, a higher education, unemployment, casual romantic relationships, and poly-drug use. On the other hand, there was no significant difference in age, frequency of drug use, HIV status, and mental health status between the participants who exhibited an intention to reduce their chemsex behavior and those who did not.

**Fig. 2:**
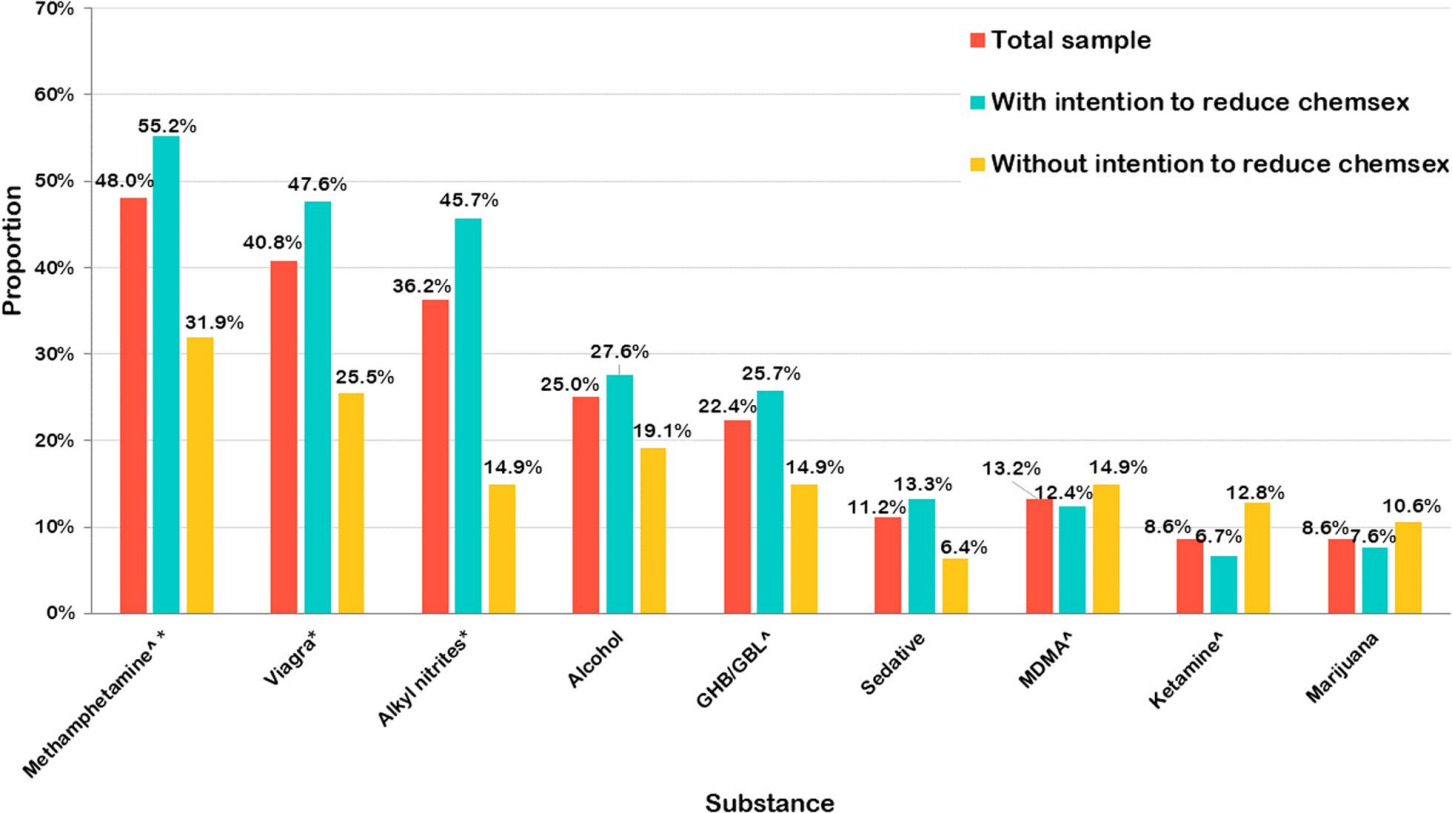
Proportion of participants with and without the intention to reduce chemsex behavior by substance used. ^**^**^Chemsex drugs, *p < 0.05

Among the chemsex drugs which were examined, methamphetamine was the most prevalent drug (48.0%), followed by GHB/GBL (22.4%), MDMA (13.2%,) and ketamine (8.6%). Figure 2 shows that a significantly larger proportion of the participants who intended to reduce their chemsex behavior used methamphetamine (55.2% vs. 31.9%), Viagra (47.6% vs. 25.5%), and alkyl nitrites (45.7% vs. 14.9%) compared to those without this intention.

### Chemsex-related mental health service utilization

Table 2 presents the bivariate associations between the baseline characteristics and service utilization. Among the 152 participants who engaged in chemsex, 23.0% had been members of a recovery group and 17.1% had visited a mental health clinic. In addition, 19.1% of all participants had used only one type of service and 10.5% had used both types.

**Table 2:** Baseline characteristics comparing participants who utilized chemsex-care services and those who did not (*N* = 152)

		Chemsex recovery groups			Mental health clinic			Types of services participants used			
	Total *N* = 152	Yes *n* = 35 (23.0%)	No *n* = 117 (77.0%)	*p*-value	Yes *n* = 26 (17.1%)	No *n* = 126 (82.9%)	*p*-value	0 *n* = 107 (70.4%)	1 *n* = 29 (19.1%)	2 *n* = 16 (10.5%)	*p*-value
	*N* (%)	*n* (%)	*n* (%)		*n* (%)	*n* (%)		*n* (%)	*n* (%)	*n* (%)	
Intention to reduce chemsex	105 (69.1)	29 (82.9)	76 (65.0)	0.044	23 (88.5)	82 (65.1)	0.019	66 (61.7)	26 (89.7)	13 (81.2)	0.008
Age < 35	114 (75.0)	24 (68.6)	90 (76.9)	0.317	17 (65.4)	97 (77.0)	0.214	83 (77.6)	21 (72.4)	10 (62.5)	0.404
Monthly income ≤ NTD$30,000	81 (53.3)	19 (54.3)	62 (53.0)	0.893	14 (53.8)	67 (53.2)	0.950	54 (50.5)	21 (72.4)	6 (37.5)	0.045
Education ≤ bachelor’s degree	123 (80.9)	30 (85.7)	93 (79.5)	0.411	20 (76.9)	103 (81.7)	0.767^b^	85 (79.4)	26 (89.7)	12 (75.0)	0.378
Unemployed	43 (28.3)	10 (28.6)	33 (28.2)	0.966	7 (26.9)	36 (28.6)	0.865	29 (27.1)	11 (37.9)	3 (18.8)	0.346
Relationship status				0.202			0.527				0.398
Single	79 (52.0)	22 (62.9)	57 (48.7)		12 (46.2)	67 (53.2)		53 (49.5)	18 (62.1)	8 (50.0)	
Regular partners	49 (32.2)	7 (20.0)	42 (35.9)		8 (30.8)	41 (32.5)		39 (36.4)	5 (17.2)	5 (31.3)	
Casual partners	24 (15.8)	6 (17.1)	18 (15.4)		6 (23.1)	18 (14.3)		15 (14.0)	6 (20.7)	3 (18.8)	
Poly-drug use	55 (36.2)	19 (54.3)	36 (30.8)	0.011	13 (50.0)	42 (33.3)	0.107	29 (27.1)	20 (69.0)	6 (37.5)	< 0.001
High frequency of substance use	25 (16.4)	13 (37.1)	12 (10.3)	< 0.001	8 (30.8)	17 (13.5)	0.061^b^	10 (9.3)	9 (31.0)	6 (37.5)	0.001
Living with HIV	39 (25.7)	17 (48.6)	22 (18.8)	< 0.001	16 (61.5)	23 (18.3)	< 0.001	14 (13.1)	17 (58.6)	8 (50.0)	< 0.001
GAD-7 and PHQ-9 scores at least one > 10^a^	45 (32.1)	10 (34.5)	35 (31.5)	0.762	10 (45.5)	35 (29.7)	0.145	29 (28.7)	12 (44.4)	4 (33.3)	0.297

NTD = New Taiwan Dollar; HIV = human immunodeficiency virus; GAD-7 = Generalized Anxiety Disorder-7; PHQ-9 = Patient Health Questionnaire-9

^a^12 Missing

^b^Yates' continuity correction (due to expected frequencies < 5)

**Fig. 3:**
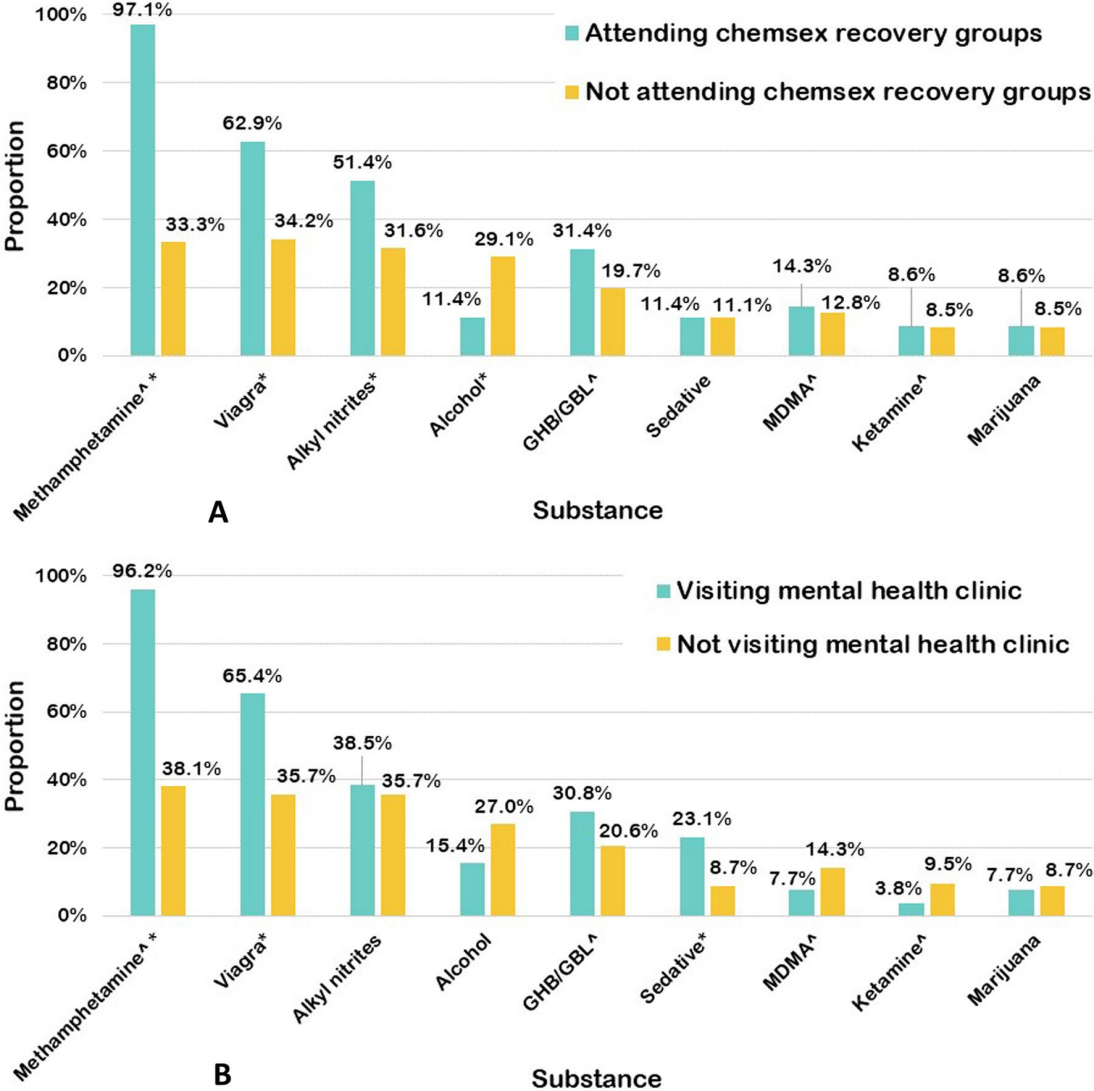
Proportion of participants using chemsex-care services or not by substance used. ^**^**^Chemsex drugs, *p < 0.05

As shown in Fig. 3A, the participants who had attended chemsex recovery groups were significantly more likely to use methamphetamine, Viagra, and alkyl nitrites, but less likely to use alcohol. Figure 3B shows that the participants who had visited a mental health clinic were significantly more likely to use methamphetamine, Viagra, and sedatives.

### Factors associated with chemsex-related mental health service utilization

The results of the univariable and multivariable regression analyses are presented in Table 3. In the univariable logistic model, joining chemsex recovery groups was significantly associated with the intention to reduce chemsex behavior (odds ratio, OR = 2.61, 95% CI 1.06–7.41), poly-drug use (OR = 2.67, 95% CI 1.24–5.85), a high frequency of substance use (OR = 5.17, 95% CI 2.09–13.04), and more likely living with HIV (OR = 4.08, 95% CI 1.82–9.25). The factors that were significantly associated with visiting a mental health clinic were the intention to reduce chemsex behavior (OR = 4.11, 95% CI 1.34–18.02), a high frequency of substance use (OR = 2.85, 95% CI 1.04–7.47), and more likely living with HIV (OR = 7.17, 95% CI 2.93–18.36). There was a positive association between the variety of services used by the participants and the intention to reduce chemsex behavior (relative risk, RR = 2.59, 95% CI 1.34–5.62), poly-drug use (RR = 1.95, 95% CI 1.18–3.23), a high frequency of substance use (RR = 2.67, 95% CI 1.54–4.47), and more likely living with HIV (RR = 3.41, 95% CI 2.07–5.69).

**Table 3:** Regression models evaluating factors associated with service utilization among GBMSM who engaged in chemsex (*N* = 152)

	Logistic regression				Poisson regression	
	Chemsex recovery groups		Mental health clinic		Types of services participants used	
	Univariable	Multivariable^a^	Univariable	Multivariable^a^	Univariable	Multivariable^a^
	OR (95% CI)	aOR (95% CI)	OR (95% CI)	aOR (95% CI)	RR (95% CI)	aRR (95% CI)
Intention to reduce chemsex	2.61 (1.06–7.41)*	2.96 (1.02–9.92)^†^	4.11 (1.34–18.02)*	4.68 (1.34–22.81)*	2.59 (1.34–5.62)*	2.31 (1.16–5.13)*
Age < 35	0.65 (0.29–1.55)	0.74 (0.26–2.13)	0.56 (0.23–1.45)	0.57 (0.20–1.66)	0.68 (0.41–1.19)	0.78 (0.44–1.42)
Monthly income ≤ NTD$30,000	1.05 (0.49–2.27)	0.65 (0.24–1.70)	1.03 (0.44–2.43)	0.58 (0.20–1.61)	1.03 (0.62–1.72)	0.69 (0.40–1.21)
Education ≤ bachelor’s degree	1.55 (0.58–4.91)		0.74 (0.28–2.22)		1.07 (0.58–2.17)	
Unemployed	1.02 (0.43–2.30)		0.92 (0.34–2.30)		0.98 (0.54–1.68)	
Relationship status (ref: single)						
Regular partners	0.43 (0.16–1.06)^†^		1.09 (0.40–2.86)		0.71 (0.38–1.28)	
Casual partners	0.86 (0.28–2.37)		1.86 (0.58–5.53)		1.16 (0.58–2.18)	
Poly-drug use	2.67 (1.24–5.85)*	3.34 (1.22–9.61)	2.00 (0.85–4.74)		1.95 (1.18–3.23)*	1.82 (0.98–3.43)^†^
High frequency of substance use	5.17 (2.09–13.04)*	6.83 (2.17–22.91)*	2.85 (1.04–7.47)*	1.83 (0.56–5.68)	2.67 (1.54–4.47)*	2.08 (1.12–3.79)*
Living with HIV	4.08 (1.82–9.25)*	2.88 (1.04–8.03)*	7.17 (2.93–18.36)*	6.46 (2.35–18.98)*	3.41 (2.07–5.69)*	2.40 (1.33–4.33)*
GAD-7 and PHQ-9						
At least one > 10	1.14 (0.47–2.67)	0.44 (0.13–1.28)	1.98 (0.77–5.01)		1.36 (0.76–2.37)	0.85 (0.46–1.53)
Missing	4.00 (1.14–14.19)*	10.18 (2.29–47.62)*	3.46 (0.82–12.91)^†^		2.55 (1.19–5.03)*	3.27 (1.42–7.10)*

OR = Odds ratio; aOR = adjusted odds ratio; RR = relative risk; aRR = adjusted relative risk; NTD = New Taiwan Dollar; HIV = human immunodeficiency virus; GAD-7 = Generalized Anxiety Disorder-7; PHQ-9 = Patient Health Questionnaire-9; ref = reference group

^a^Those variables with *p*-value < 0.05 in univariable models were then entered into the multivariable regression models, along with age and monthly income

^†^*p*-value < 0.1; *p-value < 0.05

In the multivariable logistic model, a high frequency of substance use (adjusted odds ratio, aOR = 6.83, 95% CI 2.17–22.91) and living with HIV (aOR = 2.88, 95% CI 1.04–8.03) were found to be significantly associated with being members of a chemsex recovery group, whereas the correlation between the intention to reduce chemsex behavior and joining such a recovery group was only marginally significant (aOR = 2.96, 95% CI 1.02–9.92). The use of mental health services, on the other hand, was significantly associated with both the intention to reduce chemsex behavior (aOR = 4.68, 95% CI 1.34–22.81) and living with HIV (aOR = 6.46, 95% CI 2.35–18.98). The multivariable Poisson analysis showed that the intention to reduce chemsex behavior (adjusted relative risk, aRR = 2.31, 95% CI 1.16–5.13), a high frequency of substance use (aRR = 2.08, 95% CI 1.12–3.79), and living with HIV (aRR = 2.40, 95% CI 1.33–4.33) remained significantly correlated with the types of services that the participants used.

## Discussion

This study used programmatic data obtained from an integrated sexual health services center that applies harm reduction principles to its approach to treatment. The aim was to investigate the association between the intention of people who practice chemsex to reduce their chemsex behavior and the utilization at which they use chemsex-care services. Given that its specialization in chemsex-related healthcare seems tailor-made for the GBMSM community, it is perhaps not surprising that HERO attracted quite a large number of people with the intention to reduce their chemsex behavior (69%). Our findings revealed an association between this intention and the use of mental health services. Although a large proportion of the participants expressed the intention to reduce their chemsex behavior on their first visit to the center, only 37% of them reported having used chemsex-care services, which implies that the opportunities to raise awareness and to expand the range of services available for individuals at the contemplation stage. Healthcare practitioners should help raise awareness among those who engage in chemsex regarding the consequences of their sexualized drug use and the availability of harm reduction resources. In addition, more research is needed in order to identify the barriers to access to chemsex-care services.

It is important to note that many chemsex-practicing GBMSM might not want to change their chemsex behavior or use chemsex-care services. For example, a study of chemsex-practicing MSM in the Ireland found that 75% of the participants enjoyed chemsex and felt in control of their sex lives [40]. In another study, only 19% of the MSM at nine Dutch STI clinics who engaged in chemsex intended to change their chemsex behavior [41]. On the other hand, considering that 46% of the chemsex-practicing GBMSM examined in Taiwan study exhibited signs of methamphetamine dependency [5], it seems possible that a number of these people are unaware of the harm they may potentially incur from engaging in chemsex. Furthermore, the harm resulting from their lack of awareness may be exacerbated by a lack of knowledge of the chemsex-care services that are available to them [42], making it vital that efforts at harm reduction prioritize addressing both of these issues. In order to cater for the needs of their clients who engage in chemsex, services targeting the LGBT community should also integrate such harm reduction initiatives as providing HIV testing and counseling, needle and syringe programs, and targeted information and education for chemsex and sexual health-related messages. Establishing links and collaborations between LGBT-focused health services and drug prevention and recovery services would provide a good opportunity to improve awareness of and access to healthcare.

In the current study, we found a significant relationship between the use of chemsex-care services and the intention to reduce chemsex behavior of chemsex-practicing GBMSM who visited an integrated sexual health services center. Similar results have been found for other substance-using populations, such as homeless women with substance use disorders in the USA [43]. This reinforces our finding that the willingness to change was associated with the use of services for substance use. Another study found that the motivation among cocaine users to change their drug habit correlated with their attendance at meetings of a self-help group [22].

In our study, a third of the GBMSM who engaged in chemsex used at least one chemsex-care service (i.e., joining a recovery group or visiting a mental health clinic). The utilization that we observed is similar to the finding from a study of 123 chemsex users in Germany that 30% of them had previously received psychotherapeutic treatment [34]. Another study found that 13% of MSM who had used methamphetamine in a sexual context had visited a psychotherapist, compared to only 4.1% of MSM who did not use drugs during sex [44]. However, the rate at which the chemsex-practicing GBMSM in our study used mental health services in particular was slightly higher when compared to other drug-using communities. Based on data from the National Survey on Drug Use and Health in 2013, 25.3% of American adults who were dependent on illicit drugs or alcohol had received treatment for mental health issues in the previous year [45]. The lack of agreement in the figures on the use of mental health services reported in the literature may reflect differences in the supply and demand of different kinds of services in different contexts. Furthermore, the difference between our results and those of other studies may also be attributable in part to the fact that, in contrast to the widespread use of self-reported data in much of the literature, our study based our calculation of the rate of chemsex behavior on programmatic records.

There are possible explanations for the finding that individuals who intended to reduce their chemsex behavior did not use chemsex-care services. One possibility is that they had previously used services elsewhere. For example, they may have their own psychotherapist or they may have visited psychiatrists in other clinics, which the participants in our study are not likely to have reported. However, it is also possible that they had never sought help elsewhere. People with the intention to seek help but who have not yet done so may still be at the early stages of trying to come to terms with the fact that they have a problem. Thus, they may still be weighing the pros and cons of undergoing some kind of treatment, and they may still find it difficult to discuss their drug use openly without fear of being judged [42, 46]. Some individuals may also fear that disclosing their substance use in a healthcare facility might put them at risk of legal consequences. For example, a qualitative study of MSM engaging in chemsex in Singapore revealed that local drug laws were the main barrier to them openly discussing their sexual drug use with healthcare workers, friends, and relatives [47]. The social stigma attached to drug use in general and to chemsex in particular also dissuade many from actually seeking help [42, 48]. The multifaceted stigma of engaging in chemsex is a combination of the negative views that many people have of drug use and of certain forms of sexuality, and the public rejection and marginalization of certain groups which result from various cultural and environmental factors [49]. These factors may discourage chemsex-practicing GBMSM from using appropriate health services. It has been suggested that the criminalization of the substances used during chemsex plays a major role in this stigmatization, which may lead to further discrimination against the GBMSM community [49]. Effectively addressing this issue requires collaboration among the various people who have an interest in this situation. For example, healthcare providers should work to establish trust with their clients by adopting a friendly and non-judgmental manner and by clearly letting clients know that their well-being is the priority. At the policy-making level, the reassessment of existing legal sanctions and the de-criminalization of substance use with regard to people who are using healthcare services would go a long way toward minimizing the stigma faced by people who engage in chemsex.

The findings of the present study should be considered in light of the following limitations. First, we only used the programmatic records at HERO, so we were not able to ascertain whether participants used services provided elsewhere. For example, their use of chemsex-care services may have been underestimated if they had visited a general psychiatrist who does not specialize in chemsex. This may also partially explain why we found no association between self-reports of depression and anxiety and the use of mental health services. It is possible that the participants in our study used mental health services outside HERO, which would not have factored in our analyses. Second, we did not consider the frequency of the use of chemsex-care services. Therefore, a dose–response relationship between the intention to change chemsex behavior and actually taking advantage of relevant treatment options may have existed which our study was not designed to examine. The final limitation is again related to the fact that our data were collected from one healthcare center only. This severely limits the applicability of our findings. Thus, the significant correlation that we observed between the intention of chemsex-practicing GBMSM to change their chemsex behavior and their use of health services cannot simply be generalized to the setting of other sexual health clinics or to the cases of other populations of GBMSM.

## Conclusion

Expressing the intention to reduce chemsex behavior during the baseline survey positively correlated with the use of chemsex-care services. Concern for the physical and mental consequences faced by GBMSM who practice chemsex should lend a sense of urgency to the need that currently exists for both sexual and mental health interventions and harm reduction strategies. Given that many people who engage in chemsex never seek help, public health practitioners should take steps to raise awareness among GBMSM of what harm reduction resources are available to them and how to access them when they are needed. A comprehensive health services center specializing in chemsex such as HÉROS can serve as a model for the establishment of similar service facilities in other locations. Given its presence in the community, it shows great potential for mitigating the stigma facing GBMSM and for overcoming the barriers to the access to health services for such marginalized populations. Finally, we recommend that future studies aim to gain further insight into the obstacles preventing people who engage in chemsex from seeking professional help. For example, there is a need to understand why those who intend to reduce their chemsex behavior or to get help for this behavior do not use chemsex-care services. It is also crucial to determine what kind of help these people need.

## Data Availability

Not applicable.

## Abbreviations

GBMSM: Gay and bisexual men and other men who have sex with men
HERO: The healing, empowerment, recovery of chemsex center
GHB/GBL: Gamma-hydroxybutyrate/gamma-butyrolactone
MDMA: Methylenedioxymethamphetamine
WHO: World Health Organization
ART: Antiretroviral therapy
IEC: Information, Education and Community
STI: Sexually transmitted infections
MSM: Men who have sex with men
PEP: Post-exposure prophylaxis
PrEP: Pre-exposure prophylaxis
STI: Sexually transmitted infection
LSD: Lysergic acid diethylamide
NTD: New Taiwan dollar
OR: Odds ratio
RR: Relative risk
aOR: Adjusted odds ratio
aRR: Adjusted relative risk
